# Latent burnout profile analysis in hospital doctors in Ireland

**DOI:** 10.1371/journal.pone.0308972

**Published:** 2024-08-22

**Authors:** Gordon Haire, Lucia Prihodova, Sophia Kilcullen, Blánaid Hayes

**Affiliations:** 1 Faculty of Occupational Medicine, Royal College of Physicians in Ireland, Dublin, Ireland; 2 Research Department, Royal College of Physicians in Ireland, Dublin, Ireland; University of Trieste: Universita degli Studi di Trieste, ITALY

## Abstract

**Background:**

Burnout, characterised by emotional exhaustion (EE), depersonalisation (DP) and reduced personal accomplishment (PA), is caused by chronic workplace stress. Though widely reported in doctors, variability in definitions and assessment methods render comparisons between studies challenging. Furthermore, traditional methods of reporting burnout can be misleading, focusing more on individuals than on the workplace. Various scores from Maslach Burnout inventory (MBI) have been previously reported as ‘burnout’, inflating reported prevalence. Recent research suggests using latent profile analysis (LPA), to explore the continuum from engagement to burnout, as distinct patterns of working life may contribute to different profiles.

**Aims:**

To examine the prevalence of latent burnout profiles (LBP) amongst Irish hospital doctors.

**Methods:**

LBP categorisation of MBI was applied to responses from 1610 hospital doctors from a national survey. Effort-Reward Imbalance (ERI) questionnaire and work ability score were used to measure work stress and work ability.

**Results:**

In line with LBP categorisation, the respondents were classified as follow: 23% (N = 364) Engaged; 21% (N = 332) Burnout (High EE and DP), Overextended (N = 476, 30%); 22% (N = 355) Ineffective (low PA score), 5% (N = 83) Disengaged (high DP scores). Consultants were more likely to be classified as Engaged than trainees. Those classified as Burnout were younger. Females were more likely to be classified as Overextended. Work stress was associated with Overextended, Disengaged and Burnout profiles. Insufficient workability was associated with Burnout profile.

**Conclusions:**

The use of LPA provides more nuanced exploration of the phenomenon which can be correlated with workplace features, pointing to potential interventions.

## Introduction

Burnout is an occupational phenomenon associated with chronic workplace stress. Initial work in the area focused on a syndrome of exhaustion, depersonalisation and reduced efficacy in health and social care workers. Although now recognised in the International Classification for Diseases-11 (ICD-11), it is not considered a disease state *per se* and criteria for classifying burnout in both the clinical and research setting are heterogenous [1]. Physician wellbeing has become a major worldwide concern in recent decades. In addition to an association with deleterious health effects for the individual physician, there is a recognised association with negative patient related outcomes [2]. Low rates of physician wellbeing are further associated with recruitment and retention issues, exacerbating often already strained staff resources [3–5]. As such, physician wellbeing is an occupational health, patient safety and healthcare organisational issue that requires urgent attention.

Several theories exist which conceptualise the pathway along which burnout is manifested; a clear common aetiological agent is the existence of a negative psychosocial work environment which can cause individuals to experience chronic workplace stress. Although certain individual factors can predispose to its development, without this negative workplace environment, burnout cannot develop [1, 6, 7].

The importance of the external environment and its association with potential deleterious psychological outcomes for individual workers can be seen in other areas of study. Examples include the concept of moral injury, first used to describe a PTSD-like syndrome in returned Vietnam service people who witnessed or took part in events that were contrary to their moral character or beliefs [8]. More recently, this has been considered in the setting of healthcare workers who are unable to provide care or intervention they deem to be necessary due to constraining external factors outside of their control [9]; this can result in psychological injury [10]. Another workplace model of injury highlighting the external environment is Chappel and DiMartino’s 2006 model of workplace violence; this notes the importance of the workplace environment external to the worker and its importance in contributing to psychological injury which can be independent of personal or individual factors [11].

The most commonly used tool to assess for burnout is the Maslach Burnout Inventory (MBI); the scoring and interpretation of MBI results is however varied. Rates of burnout cited in the literature vary widely depending on the utilised case definition. A 2018 systematic review of the prevalence of burnout in physicians notes that although the MBI was used in >85% of studies, quoted burnout rates range from 0–80% dependent on the definition and cut off criteria used. Indeed, recent reports of prevalence continue to conflate the presence of individual MBI components, for example isolated emotional exhaustion, with burnout [12].

Recent advances by seminal burnout researchers Maslach and Leiter advise moving away from a binary approach to burnout assessment and suggest viewing it through a prism of latent burnout profiles (LBPs) which include distinct workplace experiences between the endpoints of engagement and burnout [13]. In keeping with the experience of workplace stress as the clear aetiological agent in the development of burnout, there is an association seen between the prevalence of the differing LBPs and the distinct features of the associated work environment. The profile(s) prevalent in a workplace can potentially identify the organisational changes necessary to ameliorate its effects [14].

**Fig 1:**
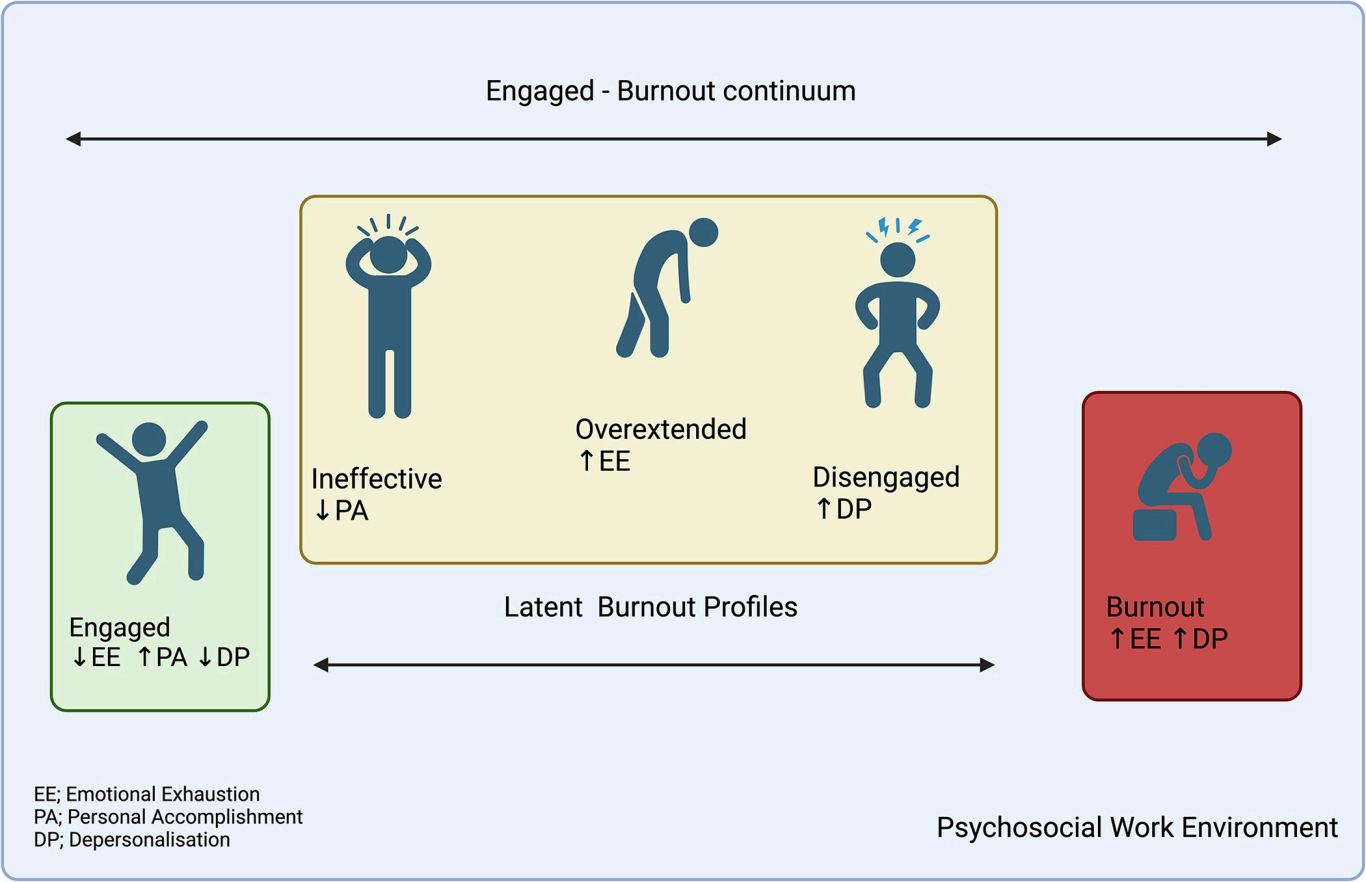
Latent burnout profiles.

Those without any significant features of burnout are considered to belong to the Engaged profile whilst in addition to a Burnout profile there exist three additional LBPs; Overextended, Disengaged and Ineffective. Classification to the relevant profile is by way of the degree of observed emotional exhaustion, depersonalisation and reduced personal efficacy (Fig 1). This classification provides a person-centred approach to assess differing workplace experiences and provide a customised method to inform relevant interventions [13].

It should be noted that although the nomenclature of LBPs has negative connotations e.g. Disengaged, Ineffective, burnout researchers are clear to point out that evidence of such changes in workers is a symptom of organisational as opposed to individual level dysfunction and that systemic and organisational change are necessary to ameliorate the effects, both directly on physicians themselves as well as on the patients for whom they care [6, 14, 15].

Given the clear association of burnout and other LBPs with a hazardous psychosocial working environment in general and workplace stress in particular, it is prudent to explore pertinent workplace factors [16]. We previously reported data from a large study on the wellbeing of hospital doctors in Ireland showing high rates of work stress and burnout, using a conservative binary classification approach [17]. We look to reanalyse the data from our original study from the perspective of LBPs, considering developments in this field of study. This paper aims to examine the prevalence of each LBP along with additional wellbeing and workplace parameters amongst hospital doctors in Ireland in order to inform appropriate future organisational interventions in the Irish healthcare environment.

## Methods

### Design

The study was a national cross-sectional survey of hospital doctors working in the Republic of Ireland.

### Participants, sample size and sampling

The sampling method including sample size calculation has been previously described in detail [18]. The participants were registered with one of nine national postgraduate medical training bodies in Ireland (the Faculty of Radiology opted out of the study) and included both consultants and doctors undertaking post graduate speciality training programmes [basic specialist trainees (BSTs) and higher specialist trainees (HSTs)].

Doctors in the study met the following inclusion criteria: fully registered with a postgraduate medical training body in Republic of Ireland and working as hospital doctor in the Irish publicly funded healthcare sector in either anaesthesia, emergency medicine (EM), medicine, obstetrics and gynaecology (O&G), ophthalmology, paediatrics, pathology, psychiatry or surgery. Only doctors working within the national publicly funded sector were included. Stratified randomisation was used.

Data were collected by way of a self-administered questionnaire. A participant information leaflet was included with the questionnaire outlining the purpose and scope of the study. The leaflet explained that the information was being gathered anonymously to ensure confidentiality and that the response to the questionnaire was indicative of consent. The data were collected by post and online from 23^rd^ April 2014-30^th^ June 2014.

### Measures

Demographic variables included participant age and sex.

### Workplace wellbeing

*Burnout* was assessed by the Maslach Burnout Inventory (MBI), which consists of three subscales assessing emotional exhaustion (EE; the feeling of being emotionally exhausted and overwhelmed by work), depersonalisation (DP; the loss of empathy and the emergence of cynicism in one’s care for others) and personal accomplishment (PA; feeling of competence in one’s work with people). Doctors were classified into one of 5 profiles according to MBI 4^th^ edition; Burnout (high EE and DP scores); Engaged (low EE and DP scores with high PA score); Overextended (high EE score only); Disengaged (high DP score only); and Ineffective (low PA score only) [19].

Additional work-related variables included grade (BST; HST; Consultant), workload [mean hours worked (MHW) per week over two consecutive working weeks in the past month], along with scales assessing their work ability, and work stress. Participants were asked about their current desire to practise medicine.

*Work ability*, the degree to which individuals are able to cope physically and mentally with the demands of work, was assessed by the Work Ability Score (WAS). This was assessed using a single item (*how would you rate your current work ability compared with your lifetime best)* with numerical response options on an 11 (0–10) point scale, where a score < 6 is considered as insufficient work ability [20].

*Work stress* was assessed using the Effort Reward Imbalance Questionnaire (ERI; 16-items; 4-point Likert scale) on three dimensions: effort (3 items, score: 3–12), reward (7 items, score: 7–28) and overcommitment (6 items, score: 6–24) perceived in one’s professional role. The effort-reward (ER) ratio ranges 0–4, where a value of 1 indicates effort reward balance while values below 1 indicate favourable working conditions and over 1 challenging working conditions and work stress [21].

The internal consistency was satisfactory for all scales (Cronbach’s α = 0.72–0.83).

### Statistical analyses

All the analyses were performed using *Statistical Package for Social Sciences* (IBM SPSS for Mac, version 28.0). Descriptive analyses were performed. Chi-squared testing of independence was performed to evaluate the relationship between LBPs and various additional personal, job- and work-related categorical variables; post-hoc z-tests on adjusted standardised residuals with Bonferroni-corrected p-values was undertaken. Differences between LBPs and continuous variables was examined using one-way ANOVA; post hoc testing using Scheffe correction methods was completed. Multinomial logistic regression was performed to identify factors associated with respective LBPs; training grades (BST, HST) were merged in this analysis.

### Ethics

The study was approved by the Royal College of Physicians of Ireland’s (RCPI) Research Ethics Committee in December 2013 (RCPI RECSAF 20).

## Results

### Sample demographics

In total, 1610 doctors participated in this study (response rate = 55%,); a further 136 participants were excluded from this analysis due to one or more missing MBI responses. Across the total sample 50% (N = 807) were male. Over half of the sample were consultants (N = 851; 53%) and the average duration of practice across the sample was 7.7 years.

Measures of personal and workplace wellbeing factors in this sample have been previously reported in detail [17, 18].

### Burnout and LBPs

One fifth of study participants met the applied criteria for Burnout (N = 332, 21%). The most common LBP was Overextended (N = 476, 30%). More than one fifth (N = 355, 22%) had isolated low personal accomplishment scores (the Ineffective profile), while one-in-twenty (N = 83, 5%) had isolated high depersonalisation scores (the Disengaged profile). Over one fifth (N = 364, 23%) are classified as Engaged without evidence of any burnout parameter.

Chi-squared testing of independence was performed to evaluate the relationship between LBPs and various additional personal, job- and work-related categorical variables.

The relationship between LBPs and both **age** [χ^2^ (20, *N =* 1601 = 150.789, *p* = <0.001)] and **sex** [χ^2^ (4, *N =* 1608 = 13.310, *p* = 0.010)] was significant (Table 1). Post hoc z-testing on adjusted standardised residuals with Bonferroni corrected p-values revealed that significantly greater numbers of doctors in the 26–30 year age group fitted the Burnout profile, [adjusted *p* < .001 (Bonferroni adjusted threshold for significance was 0.001)]; furthermore, a significant association between female sex and the Overextended profile was shown [p<0.005 (Bonferroni adjusted threshold for significance was 0.005)].

**Table 1:** Personal and work-related characteristics amongst latent burnout profiles.

		All N = 1610 (100)	Engaged N(%) = 364 (20.8)	Ineffective N (%) = 355 (20.3)	Overextended N (%) = 476 (27.3)	Disengaged N (%) = 83 (4.8)	Burnout N (%) = 332 (19.0)	χ^2^
		**N (%)**	**N (%)**	**N (%)**	**N (%)**	**N (%)**	**N (%)**	
Sex								13.310 **
	Male	807 (50.2)	195 (53.7)	170 (48.0)	213 (44.7)	50 (60.2)	179 (53.9)	
	Female	801 (49.8)	168 (46.3)	184 (52.0)	263 (55.3)^**B**^	33 (39.8)	153 (46.1)	
Age								150.789 ***
	< = 25	37 (2.3)	2 (0.6)	6 (1.7)	9 (1.9)	5 (6.1)	15 (4.5)	
	26–30	298 (18.6)	39 (10.8)	57 (16.2)	66 (13.9)	29 (35.4)	107 (32.3)^**B**^	
	31–40	502 (31.4)	97 (26.9)	108 (30.7)	160 (33.7)	30 (36.6)	107 (32.3)	
	41–50	427 (26.7)	112 (31.0)	102 (29.0)	140 (29.5)	9 (11.0)	64 (19.3)	
	51–60	286 (17.9)	87 (24.1)	65 (18.5)	92 (19.4)	7 (8.5)	35 (10.6)	
	>60	51 (3.2)	24 (6.6)	14 (4.0)	8 (1.7)	2 (2.4)	3 (0.9)	
Grade								119.971***
	Consultant	851 (52.9)	248 (68.1)^**B**^	199 (56.1)	273 (57.4)	20 (24.1)	111 (33.4)	
	HST	402 (25.0)	65 (17.9)	85 (23.9)	111 (23.3)	30 (36.1)	111 (33.4)	
	BST	357 (22.2)	51 (14.0)	71 (20.0)	92 (19.3)	33 (39.8)	110 (33.1)^**B**^	
Current work-ability								*179*.308 ***
	Insufficient (WAS <6)	508 (29.1)	41 (11.3)	67 (18.9)	207 (43.5)^**B**^	14 (16.9)	159 (47.9)^**B**^	
	Sufficient (WAS >6)	1238 (70.9)	323 (88.7)	288 (81.1)	269 (56.5)	69 (83.1)	173 (52.1)	
Work stress								159.664 ***
	Absent (ERI Ratio <1)	265 (17.8)	114 (34.2)	91 (27.3)	28 (6.4)	18 (23.1)	14 (4.5)	
	Present (ERI Ratio >1)	1225 (82.2)	219 (65.8)	242 (72.7)	409 (93.6)^**B**^	60 (76.9)	295 (95.5)^**B**^	
Current desire to practise medicine								272.849***
	Very strong	402 (25.1)	170 (46.7)	82 (23.2)	90 (19.0)	20 (24.1)	40 (12.1)^**B**^	
	Strong	723 (45.1)	161 (44.2)	198 (56.1)	197 (41.6)	41 (49.4)	126 (38.2)	
	Lukewarm	359 (22.4)	30 (8.2)	84 (18.1)	145 (30.7)	20 (24.1)	100 (30.3)	
	Weak	61 (3.8)	2 (0.5)	7 (2.0)	20 (4.2)	1 (1.2)	31 (9.4)	
	Regret becoming a doctor	58 (3.6)	1 (0.3)	2 (0.6)	21 (4.4)	1 (1.2)	33 (10.0)	
Participates in “on-call” roster								1.328 ^NS^
	Yes	1457 (90.7)	334 (91.8)	317 (89.5)	431 (90.9)	74 (89.2)	301 (90.9)	
	No	149 (9.3)	30 (8.2)	37 (10.5)	43 (9.1)	9 (10.8)	30 (9.1)	
Full or part time work								5.393 NS
	Full time	1545 (96.0)	346 (95.1)	337 (95.2)	456 (95.8)	82 (98.8)	324 (97.6)	
	Part time	64 (4.0)	18 (4.9)	17 (4.8)	20 (4.2)	1 (1.2)	8 (2.4)	
Workload		***AM (SD)***	***AM (SD)***	***AM (SD)***	***AM (SD)***	***AM (SD)***	***AM (SD)***	***F***
	*Mean hours worked*	*57*.*0 (15*.*1)*	*54*.*1 (15*.*0)*	*54*.*4 (13*.*7)*	*47*.*8 (15*.*1)*^**B**^	*58*.*7 (12*.*8)*	*61*.*9 (15*.*6)*^**B**^	*15*.938 ***

All associations tested by cross tabulation, except for workload which is tested by ANOVA

* p<0.05

** p<0.01

***p<0.001, NS: p>0.05

B: statistically significant post hoc testing

AM: arithmetic mean

SD: standard deviation

There is a significant association between **employment grade** and burnout profiles [*X2* = 119.971, *df* = 8, p < .001]. Post-hoc testing revealed that consultants were significantly more Engaged (29%) than BST (14%) or HST (16%) respondents, and there were significantly more consultants represented in the Engaged versus the Disengaged (16%) and Burnout (14%) profiles. Significantly greater numbers of BSTs than consultants fitted the Burnout profile (31% v. 13%), adjusted *p* < .003 (Bonferroni adjusted threshold for significance was 0.003).

There is a significant relationship between **workplace stress**, measured as **ERI,** and LBPs [χ^2^ (4, *N =* 1490 = 159.664, *p* = <0.001)]. Post hoc analysis showed there are significantly more practitioners with ERI in the Burnout (96%) and Overextended (94%) profiles, than in the Engaged (66%) and Ineffective (73%) profiles [adjusted p<0.0001 (Bonferroni adjusted threshold for significant 0.005)].

Chi-square testing showed a significant association between LBP and ongoing **desire to practise medicine** [X2 = 272.849, df = 16, p < .001]. Post-hoc testing revealed a significantly smaller number of doctors with the Burnout profile (12%) expressed very strong desire to practise medicine than doctors with an Engaged (47%), Ineffective (23%) or Disengaged (24%) profile, adjusted *p* < .002 (Bonferroni adjusted threshold for significance was 0.002).

Concerning **work ability**, once again there is a significant association with LBPs by chi-square testing [χ^2^ (4, *N = 1610* = 179.308, *p* = <0.001)]. Post hoc analysis reveals significantly more doctors with insufficient workability in the Burnout (48%) and Overextended (44%) profile with lower rates of insufficient workability in the Engaged (11%) and Ineffective (19%) profiles, adjusted P <0.0001 (Bonferroni adjusted threshold 0.005).

There was no significant association between LBPs and whether on-call work was undertaken [χ^2^ (4, *N = 1606* = 1.328, *p* = 0.857)] or whether work was full time or part time[χ^2^ (4, *N = 1609* = 5.393, *p* = 0.249)].

One-way ANOVA examining the relationship between LBPs and MHW [at the 0.05 level, *F*(4, 1563 = 15.938, *p* = <0.001)] is significant. Scheffe’s post hoc testing for multiple comparisons showed that the MHW was significantly higher for the Overextended (p = 0.012, 95% CI 0.92–6.95 additional MHW) and Burnout profile (p<0.001, 95% CI 4.37–11.35 additional MHW) compared to the Engaged profile; there is no significant difference between MHW for the Ineffective (p = 0.999, 95% CI -3.73 to +3.14 MHW) and Disengaged (p = 0.162, 95% CI -10.21 to +0.95 MHW) profiles compared to the engaged profile.

### Multinomial logistic regression

A multinomial logistic regression model (Table 2), examining 6 personal and work-related wellbeing factors associated with LBPs, with the Engaged profile as the comparator, correctly predicted 59% of the Engaged profile, 17% of the Ineffective profile, 56% of the Overextended profile, 1% of the Disengaged profile and 47% of the Burnout profile; it correctly predicted the LBP 43% of the time.

**Table 2:** Multinomial logistic regression of personal and work-related factors and latent burnout profiles.

LBP^a^	Overextended			Disengaged			Burnout			Ineffective		
	Wald	Exp(B)	95% CI Exp(B)	Wald	Exp(B)	95% CI Exp(B)	Wald	Exp(B)	95% CI Exp(B)	Wald	Exp(B)	95% CI Exp(B)
Sex^b^	0.052	0.961^NS^	0.682–1.354	11.973	0.374***	0.214–0.653	13.563	0.477***	0.322–0.707	0.004	0.989^NS^	0.706–1.385
Grade^c^	1.281	1.212^NS^	0.869–1.691	7.741	2.068**	1.239–3.449	3.015	1.396^NS^	0.958–2.033	0.562	1.13^NS^	0.821–1.554
Workplace stress (ERI)	53.252	6.153***	3.777–10.024	4.817	2.01*	1.078–3.748	44.799	8.958***	4.714–17.023	1.332	1.233^NS^	0.864–1.761
Workability	39.906	0.258***	0.17–0.393	0.667	0.74^NS^	0.358–1.526	41.034	0.221***	0.139–0.351	3.517	0.648^NS^	0.412–1.02
Workload (MHW)	7.577	1.016**	1.005–1.028	1.385	1.012^NS^	0.992–1.031	17.691	1.028***	1.015–1.042	0.183	1.003^NS^	0.991–1.014
Current desire to practise medicine	75.731	2.724***	2.174–3.414	23.811	2.311***	1.651–3.236	113.599	3.742***	2.936–4.77	35.924	2.001***	1.595–2.51

* p<0.05

** p<0.01

***p<0.001, NS: p>0.05

a Reference category is: Engaged.

b Male sex

c Consultant

ERI: Effort reward imbalance

MHW: Mean hours worked

The presence of a lower current desire to practise medicine [(Exp(B) = 2.001, SE = .116, p = <0.001) was significantly associated with the Ineffective profile.

For the Overextended profile, the presence of work stress, measured as ERI, [(Exp(B) = 6.513, SE = 0.249, p = <0.001)]; insufficient workability [(Exp(B) = 0.258, SE = .214, p = <0.001)]; a lower current desire to practise medicine [Exp(B) = 1.878, SE = 0.138, p = <0.001)] and a larger workload, measured as MHW, [(Exp(B) = 1.016, SE = .006, p = 0.006)] were significantly associated.

Considering the Disengaged profile, male sex [(Exp(B) = 0.356, SE = 0.295, p = <0.001)]; the presence of workplace stress [(Exp(B) = 2.010, SE = 0.318, p = 0.0.028]; lower employment grade [(Exp(B) = 2.068, SE = 0.261, p = 0.005)] and a lower current desire to practise medicine [(Exp(B) = 2.311, SE = 0.172, p = <0.001)] were significantly associated.

Finally, looking at the Burnout profile, male sex [(Exp(B) = 0.477, SE = .201, p = <0.001)]; the presence of work stress [(Exp(B) = 8.958, SE = 0.328, p = <0.001)]; insufficient workability [(Exp(B) = 0.221, SE = 0.236, p = <0.001); a lower current desire to practise medicine [Exp(B) = 3.742, SE = 0.124, p = <0.001)] and a larger workload [(Exp(B) = 1.028, SE = 0.007, p<0.001)] were significantly associated.

## Discussion

A reanalysis of this large cohort of hospital doctors working in Ireland shows high rates of Burnout along with the Overextended and Ineffective latent profiles; relatively few study participants report no features of burnout, fitting the so-called Engaged profile. Such high rates of burnout and LBPs suggests significant workplace challenges and points to issues with the external workplace environment. Indeed, there are a number of significant associations between relevant workplace factors and LBPs with a particular picture of deleterious workplace conditions associated with the Burnout and Overextended profiles. There are further associations between these profiles relevant to recruitment and retention, namely low levels of both ongoing desire to practise medicine and work ability.

The most common LBP seen in our cohort is the Overextended profile. This profile is typified by high rates of emotional exhaustion; such a picture suggests a requirement for intervention and solutions that focus on reducing workload as well as reducing fatigue and its effects.

Despite improvements in recent years, the Irish healthcare system has low levels of consultant doctors by international standards [22]. Increasing the number of consultant physicians may act to reduce workload on current practitioners although it is noted that there are high levels of consultant vacancies, as well as a high number of vacancies across other clinical healthcare professions, in the healthcare system in Ireland [23].

Ireland also has a relatively high proportion of non-consultant hospital doctors (NCHDs; non-consultant hospital doctors comprising BSTs, HSTs and those not in training) working in the hospital system [19]. NCHDs in the Irish healthcare system complain of a large volume of routine basic clinical and non-clinical or administrative tasks which could be undertaken by others [24–26]. The role of physician’s assistant is nascent in the Irish context; this could be a potential intervention to aid in reducing workloads and related pressures on doctors in the Irish healthcare system although other health systems with such clinical supports also exhibit high rates of burnout [12, 27].

Given a relatively high proportion of the Ineffective profile, with high levels of reduced personal efficacy, interventions to increase engagement and reduce alienation are important. Fostering psychologically safe workplaces may act as an efficacious intervention here [28]. A combination of the above measures is necessary for those fitting the burnout profile.

Effort reward imbalance, a validated measure of workplace stress, is associated with the Overextended and Burnout profiles. A 2022 metanalysis of ERI in physicians showed a markedly wide ranging ERI dependent on both work location and other work related variables. There are high rates of ERI in Ireland when compared to other locations included in this metanalysis and this may explain the high proportions of practitioners seen in each of these profiles here [29].

Younger doctors in this cohort and those in lower employment grades are more likely to exhibit Burnout or LBPs; this may be explained by the *healthy worker effect* [30]. This may indicate the need for early-stage career interventions to prevent potential loss of personnel from the hospital system.

Burnout rates among doctors vary widely in published literature; the heterogenous definition and applied case criteria can make comparison between studies difficult [12]. This can be illustrated by way of example by considering a 2023 study by Crudden et al concerning burnout amongst Irish consultant doctors; here caseness for burnout is considered a single score elevation of either EE or DP yielding a noted burnout rate of 46% [31]. Indeed, as noted above, Rotenstein et al, in a 2018 systematic review of burnout amongst physicians, note rates ranging from 0–80% dependent on the burnout definition used [12].

Other studies amongst healthcare workers looking specifically at MBI LBPs show a different picture to that seen in our sample. A 2021 study of 150 doctors, nurses and other allied healthcare professionals in France by Boulier et al showed Disengaged as the most prevalent profile (37%); there were higher rates of the Engaged profile (35%) and lower rates of Overextended (16%) and Burnout (8%) than seen in our cohort [32]. Giusti et al report a pre-pandemic assessment of LBPs amongst 337 Italian nurses showing higher rates of Engaged (67%) and similar rates of Ineffective (15%) and Burnout (18%) with a notable absence of Overextended and Disengaged profiles [33]. Comparison here highlights the prevalence of the Overextended profile amongst our study participants which is not replicated in healthcare workers in other European based settings; this profile’s association with high workloads in our study may indicate that this is a particular problem in our health service.

### Strengths and limitations

To our knowledge, this is the largest study to date of wellbeing of hospital doctors working in a single health system and the application of recent research developments to assign LBPs to this large existing cohort highlights work factors associated with profiles along the engagement-burnout continuum which may be useful to inform relevant organisational interventions.

A limitation of the study is that data collection occurred in 2014, prior to the onset of the Covid-19 pandemic, which itself had an impact on the psychosocial healthcare work environment [34–36]. Thus, negative wellbeing parameters reported here may be an underestimate.

Using the LBP method of MBI interpretation results in a smaller number of participants in each profile compared to using a binary classification approach reducing the statistical precision of the study findings. Comparing parameters across multiple groups increases the likelihood of type 1 error; this is controlled in this study with conservative post hoc analysis [37]. The cross-sectional nature of this study does not allow inference for causation; longitudinal study here would be informative.

As NCHDs not in training were not linked to a specialist post-graduate college at the time of data collection, their experience is not captured or reflected in these results.

### Implications for future research

As noted, as this data predates the COVID-19 pandemic it would be prudent to examine the prevalence of burnout, LBPs and associated workplace parameters in a post pandemic healthcare environment. A focus on the noted association in our study of Overextended and Burnout profiles with certain negative workplace factors, through both qualitative and quantitative methods, is likely to be particularly informative.

## Conclusion

There are high rates of Burnout and latent burnout profiles in doctors working in the Irish healthcare system, suggesting significant issues with the external workplace environment. This data provides a robust pre-pandemic assessment of the workplace factors associated with respective LBPs. Relevant organisational interventions are necessary to ameliorate such high rates of individuals seen beyond the engaged profile in the engaged to burnout continuum; without such interventions there is a risk not just to the practitioner, but also those they care for and the wider healthcare system.
